# Transcriptome analysis highlights the influence of temperature on hydrolase and traps in nematode-trapping fungi

**DOI:** 10.3389/fmicb.2024.1384459

**Published:** 2024-05-07

**Authors:** Hanqi Jia, Rui Xia, Ruizhi Zhang, Guanjun Liang, Yuting Zhuang, Yantao Zhou, Danlei Li, Feng Wang

**Affiliations:** ^1^Key Laboratory of Alien Forest Pest Detection and Control-Heilongjiang Province, School of Forestry, Northeast Forestry University, Harbin, China; ^2^Center for Biological Disaster Prevention and Control, National Forestry and Grassland Administration, Shenyang, China; ^3^Key Laboratory of Forest Plant Ecology, Ministry of Education, School of Forestry, Northeast Forestry University, Harbin, China; ^4^State Key Laboratory of Tree Genetics and Breeding, Northeast Forestry University, Harbin, China

**Keywords:** nematode-trapping fungi, *Arthrobotrys cladodes*, traps, *Bursaphelenchus xylophilus*, RNA-seq

## Abstract

Pine wilt disease caused by *Bursaphelenchus xylophilus* poses a serious threat to the economic and ecological value of forestry. Nematode trapping fungi trap and kill nematodes using specialized trapping devices, which are highly efficient and non-toxic to the environment, and are very promising for use as biological control agents. In this study, we isolated several nematode-trapping fungi from various regions and screened three for their high nematocidal efficiency. However, the effectiveness of these fungi as nematicides is notably influenced by temperature and exhibits different morphologies in response to temperature fluctuations, which are categorized as “NA,” “thin,” “dense,” and “sparse.” The trend of trap formation with temperature was consistent with the trend of nematocidal efficiency with temperature. Both of which initially increased and then decreased with increasing temperature. Among them, *Arthrobotrys cladodes* exhibited the highest level of nematocidal activity and trap formation among the tested species. Transcriptome data were collected from *A. cladodes* with various trap morphologies. Hydrolase activity was significantly enriched according to GO and KEGG enrichment analyses. Eight genes related to hydrolases were found to be consistent with the trend of trap morphology with temperature. Weighted gene co-expression analysis and the Cytoscape network revealed that these 8 genes are associated with either mitosis or autophagy. This suggests that they contribute to the formation of “dense” structures in nematode-trapping fungi. One of these genes is the serine protein hydrolase gene involved in autophagy. This study reveals a potentially critical role for hydrolases in trap formation and nematocidal efficiency. And presents a model where temperature affects trap formation and nematocidal efficiency by influencing the serine protease *prb1* involved in the autophagy process.

## 1 Introduction

The pine wood nematode (PWN, *Bursaphelenchus xylophilus*) is an obligate endoparasitic nematode of Pinaceae species that causes pine wilt disease (PWD), which is native to North America and causes severe damage to pine trees, inducing substantial economic and ecological losses (Zhang et al., 2022; Shi et al., 2023). PWD was first discovered in 1982 at Purple Mountain in Nanjing, China. In recent years, the disease has spread from south to north in China and has extended to Liaoning and Jilin provinces in the northeast, and the situation is becoming increasingly serious (Chen et al., 2021). Therefore, there is an urgent need to deal with *B. xylophilus* to protect forests. For decades, control of *B. xylophilus* has relied primarily on chemical nematicides, although natural enemy control and resistant crop cultivation have been complementary methods. However, because of their notorious toxicity to wildlife and human health, available chemical nematicides are being withdrawn from use, so it is urgent to find new, environmentally friendly, and effective management strategies to control PWN (Li et al., 2015).

Nematode-trapping fungi are a group that parasitize, capture, colonize, and produce toxins to kill nematodes. It is estimated that nematode-trapping fungi are a unique group of carnivorous microorganisms that diverged from saprophytism around 419 million years ago. To survive in nitrogen-deficient environments, they develop specialized predation devices such as sessile adhesive knobs, constriction rings, and adhesive networks that provide additional sources of nitrogen and nutrients to tackle nutrient depletion by trapping and digesting nematodes once in contact with them (Barron, 1977; Belder et al., 1993; Yang et al., 2012; Quijada et al., 2020). The trapping structures of nematode-trapping fungi are not just tools for capturing and infecting nematodes, but also an important indicator of the shift in lifestyle from saprophytic to parasitic. This remarkable characteristic prompted researchers to consider the role of these fungal biocontrol agents in preventing parasitic nematodes in plants and animals (Andersson et al., 2014; Su et al., 2017).

As nematode parasites and important natural enemies, they make important contributions to the regulation of nematode populations in different environments and reduce economic losses. Environmental pollution, drug resistance, and drug residues associated with chemical control were also avoided (Andersson et al., 2014; Chen et al., 2020). Therefore, nematode-trapping fungi have recently become the most researched natural nematode antagonists (Jiang et al., 2017). They are also valuable tools for managing parasitic nematodes in plants and animals (Devi, 2018; Mendoza-de Gives et al., 2018) and are considered efficient nematode control methods in livestock and agriculture (Ocampo-Gutiérrez et al., 2021; Quevedo et al., 2021; Cristóbal-Alejo et al., 2023). It’s projected that parasitic nematodes lead to over $80 billion in yearly economic damages (Rodríguez-kábana and Canullo, 1992; Guillermo and Hsueh, 2018), encompassing those that result in weight reduction in livestock (Grønvold et al., 1993) and diseases of plants transmitted through soil (Jones et al., 2013). Within the agricultural sector, the traditional method for biologically managing parasitic nematodes in soil-based plant diseases involves directly introducing nematode-trapping fungi into the soil. For livestock, administering nematodes in pellets with these fungi orally to cattle and sheep has been more effective, leading to a 68.7% decrease in larval numbers (Oliveira et al., 2018) and 83.5% reduction (Rodrigues et al., 2020).

Previous studies showed relatively limited research and applications related to controlling *B. xylophilus* by nematode-trapping fungi. Although there is some effect of them on the control of *B. xylophilus*, most of them come from soil and animals, thus these fungi have difficulty adapting to the environment in trees (Zhang et al., 2021b). In this study, several nematode-trapping fungi were isolated and characterized in different regions. Of these, *A. cladodes*, *A. conoides*, and *A. robusta* were more effective against *B. xylophilus*. Next, we assessed the insecticidal activity and trap formation of the three fungi at different temperatures. They have been observed to respond to temperature in unison. All of them increased first and then decreased with the increase of temperature. Ultimately, our screening led to the discovery of a highly nematocidal *A. cladodes* strain, unveiling its nematocidal process to assess how effective nematode-trapping fungi are in managing *B. xylophilus*.

Previous studies have found that maintaining an active nematophagous state in nematode-trapping fungi is related to many factors. The results of different treatment bioassays performed to evaluate fungal responses during nematode-trapping fungi-nematode interactions showed that they affected the number of traps generated by nematode-trapping fungi and the rate at which they trapped nematodes, such as cornmeal agar (CMA) and potato dextrose agar (PDA) (Hsueh et al., 2013), direct physical contact and non-direct contact, living and dead nematodes (Wang et al., 2018), and carbon and nitrogen sources (Migunova and Byzov, 2005; Anan’ko and Teplyakova, 2011). Temperature and oxygen have an effect on the number of traps webs produced by nematode-trapping fungi (Zerong, 1999). However, the molecular mechanism of nematocidal effect and trap formation by temperature and the expression level of related genes are not clear. Our observations show that trap formation is significantly correlated with nematocidal activity and displays varied morphological characteristics at different temperatures. There was no significant difference in mycelial status after adding nematode-induced mycelium at less than 18°C. At 22°C, the trap did not form a closed circle, and the three-dimensional structure was “thin”; at 28°C, the three-dimensional structure was “dense,” and the nematodes were easily attracted and trapped by the trap and challenged to break free, increasing the nematode’s killing efficiency. At 32°C, an unstable, “sparse” three-dimensional structure makes it more difficult to trap nematodes. However, previous studies have not emphasized this phenomenon, resulting in the limitation of nematode-trapping fungi in production applications, thus ignoring the high efficiency of nematode-trapping fungi in killing nematodes. It is necessary to study the ability of nematode-trapping fungi to form traps under different environmental conditions.

In the present study, we collected transcriptomic data of *A. cladodes* at different temperatures, employing trap morphology as a critical indicator. Weighted gene co-expression network analysis (WGCNA) was used to explore the complex relationship between genes and phenotypes, assisting in identifying the essential functions of genes in modules associated with trap formation. The results show that distinct traps are structurally capable of producing hydrolases, and the hydrolase genes involved in trap formation and temperature variation are considered potential targets for nematode-trapping fungi.

## 2 Materials and methodology

### 2.1 Sample preparation

*Arthrobotrys cladodes* isolated from the xylem of dead and diseased *Pinus koraiensis* in Fushun City, Liaoning Province, China, in May 2021 (125° 28′ E 44° 28′ N). *A. conoides* isolated from the xylem of dead and diseased *Pinus massoniana* in Pan’an City, Zhejiang Province, China, in May 2021 (120° 24′ E 28°58′ N). *A. robusta* was isolated from the soil in Harbin City, Heilongjiang Province, China, in May 2022 (125° 42′ E 44° 04′ N). Meanwhile, the same method was used to isolate nematode-trapping fungi from healthy pine trees (Zhang et al., 2021a). It was identified by molecular and morphological characterization, respectively. They were inoculated in plates of LCMA at the late stage of mycelial growth with the addition of *B. xylophilus* as a treatment group and without *B. xylophilus* as a control group. Then, the three nematode-trapping fungi were incubated at 18–32°C. Once the mycelium appeared to have a typical trap structure, the trapping area was measured and recorded using ImageJ software. Based on the temperature associated with different morphological structures of the traps (“NA” −18°C, “thin” 22°C, “dense” −28°C, “sparse” −32°C), the treatment groups were designated as “NA,” “thin,” “dense,” and “sparse”. Three replicates of the test were performed. The death of nematodes after 36 h was observed and recorded, and the nematicidal efficiency was calculated according to the formula in Eq. 1.


(1) $$\text{E}=\left(1-\frac{{\text{N}}_{\text{r}}}{{\text{N}}_{\text{i}}}\right)\times 100\%$$

E, nematocidal efficiency; N_r_, remaining number of nematodes; N_i_, initial number of nematodes.

### 2.2 Differentially expressed gene (DEG) library preparation and sequencing

#### 2.2.1 Analysis of raw data

*Arthrobotrys cladodes* inoculated with *B. xylophilus* suspension were treated as ABXH18, ABXH22, ABXH28, and ABXH32as the treatment group and *A. cladodes* inoculated with equal amounts of sterile water were named as NBXH18, NBXH22, NBXH28, and NBXH32 as the CK group. Total RNA was extracted separately using TRIzol (Invitrogen) (Pandit et al., 2017). Three replicates of each sample were performed. A total of 3 μg of high-quality RNA per sample was used as the input material for the RNA sample preparations. Transcriptome sequencing was performed on an Illumina HiSeq 150 platform (BGI, Shenzhen, China). concentration of total RNA, RNA integrity number (RIN), 28S/18S and fragment size were assessed by the RNA Nano 6000 Assay Kit for the Agilent 2100 Bioanalyzer System (Agilent, USA). the purity of RNA was determined by a NanoDrop spectrophotometer (Termo Scientifc, USA). Each RNA sample was fragmented as a template and reverse transcribed using random primers to obtain cDNA for library construction. The construction of the libraries and sequencing was performed on an Illumina HiSeq 150 platform (BGI, Shenzhen, China). The raw sequencing reads obtained from sequencing run were filtered for minimum length of 50 bases and mean quality score *Q* ≥ 15 using SOAPnuke (v1.6.5).^1^ The reads of induced samples were mapped to *B. xylophilus* whole genome using HISAT2 and mapped reads were eliminated. The clean reads were mapped to reference sequences using Bowtie2 (v2.4.5)^2^ (Dewey and Bo, 2011; Langmead and Salzberg, 2012; Pandit et al., 2017). The matched reads were calculated and normalized to fragments per kilobase per million mapped fragments (FPKM) using RSEM (v1.2.12).^3^ Identification of differentially expressed gene.

With a view to linking the induction of *B. xylophilus* to differences in gene expression in *A. cladodes*. Multiple hypothesis test corrections were performed according to the *P*-values of the tests, and the domain value of the *P*-value was determined by controlling for the FDR (False Discovery Rate). Among them, log_2_(Treatment/CK) ≥ 1 was defined as up-regulation of expression, log_2_(Treatment/CK) ≤ −1 was defined as down-regulation of expression, and −1 < log_2_(Treatment/CK) < 1 was defined as no significant change in expression. Ten genes were randomly selected for RT-PCR to verify the reliability of the transcriptome data. All primer sequences are shown in Supplementary Table 2. The RT-PCR results were normalized (log_2_[fold-change]) with the *β-actin* and *18S ribosomal RNA* as reference genes. The relative expression levels of each gene were analyzed by the 2^–ΔΔCT^ method, and each reaction involved three independent repeated tests (Chen et al., 2021; Zhang et al., 2022).

#### 2.2.2 Construction of the weighted gene co-expression network analysis (WGCNA)

Weighted gene co-expression network analysis (WGCNA) constructed with an R package identifies highly synergistic gene sets and is used to describe complex relationships between genes and phenotypes in different samples (Langfelder and Horvath, 2008). Clustering of all samples based on the expression of all genes to analyze sample relationships, the dynamic tree cut method was used to cluster genes and divide them into modules; the gene clustering tree was constructed based on the correlation of gene expression; the minimum number of genes in a module was set to 30; and the merging threshold of similar modules was 0.8. The modules associated with the traits were analyzed by module eigengene (ME). Following multiple testing corrections, GO (Ashburner et al., 2000) terms and KEGG pathways that met this condition were considered significantly enriched pathways with a *P*-value < 0.05 as a threshold.

#### 2.2.3 Validation of gene expression by RT-PCR

RT-PCR was used to determine the expression of 8 hydrolase genes in *A. cladodes* with only *B. xylophilus* added at different temperatures and the primers were designed using Primer Premier 5. All primer sequences are shown in Supplementary Table 2. According to the instructions of the manufacturer, the Hieff UNICON^®^ Universal qPCR SYBR Green Master Mix (Yeasen, Shanghai, China) and the Stratagene Mx3000P Qpcr system (Agilent Technologies, Santa Clara, California, USA) were used for RT-PCR. The RT-qPCR results were normalized as log_2_(ABXHX/ABXH18)-fold change with the *β-actin* and *18S ribosomal* as reference genes, which has no significant diference in expression level at each stage. The relative quantification method was used to calculate the data, and three independent replicates of each reaction were performed (Chen et al., 2021; Zhang et al., 2022).

## 3 Results

### 3.1 Identification of nematode-trapping fungi and characteristics of trap formation

**FIGURE 1:**
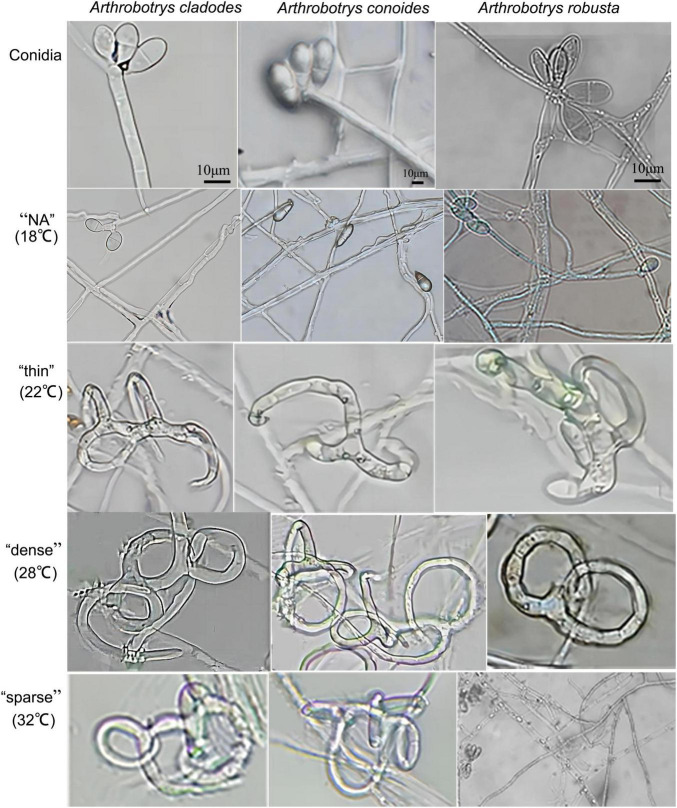
Conidia of three nematode-feeding fungi and the trap structures they form at the corresponding temperatures. From left to right: *A. cladodes*, *A. conoides*, and *A. robusta*; from top to bottom: conidia, “NA,” “thin,” “dense,” and “sparse” trap structures (NA indicates no trap was formed).

*Arthrobotrys cladodes* and *A. conoides* were isolated from *P. koraiensis* and *P. massoniana* infected with *B. xylophilus*, respectively. *A. robusta* was isolated from the soil. However, there were no nematode-trapping fungi isolated from healthy pines groups. This indicates that nematode-trapping fungi have a very strong dependence on the *B. xylophilus* and have tracking and lagging inhibition effects. This is consistent with the results of Zhang’s study (Zhang et al., 2021a). Colonies of *A. cladodes* were white on CMA medium, later producing pink pigment; conidiophores erect, unbranched, or produced 1–2 branches, apically irregularly inflated; conidia borne on short, slender dentate peduncles; conidia [17.2–25.7 × 6.8–11 μm (*n* = 50)] oblong-obovate to oblong-ellipsoid, apically rounded, a septum located in the middle. Colonies of *A. conoides* were white on CMA medium, later producing a slight yellowish color; conidiophores erect, unbranched, solitary, colorless, apically tapering, apically with 2 to 7 conidia borne on verruculose nodes; conidia [17.3–23.3 × 8.7–10.8 μm (*n* = 50)] were colorless, smooth, obconical, and constricted at the septa. Colonies of *A. robusta* white on CMA medium; conidiophores erect, often branched, slightly expanded apically; up to 5–6 conidia produced on short, slenderly dentate peduncles; conidia [18.3–26.3 × 9.7–12 μm (*n* = 50)] obovate, with a segregation near the middle. *A. cladodes*, *A. conoides*, and *A. robusta* all prey on nematodes with three-dimensional fungal webs (Figure 1). The results showed significant differences in the traps’ morphology at different temperatures.

**FIGURE 2:**
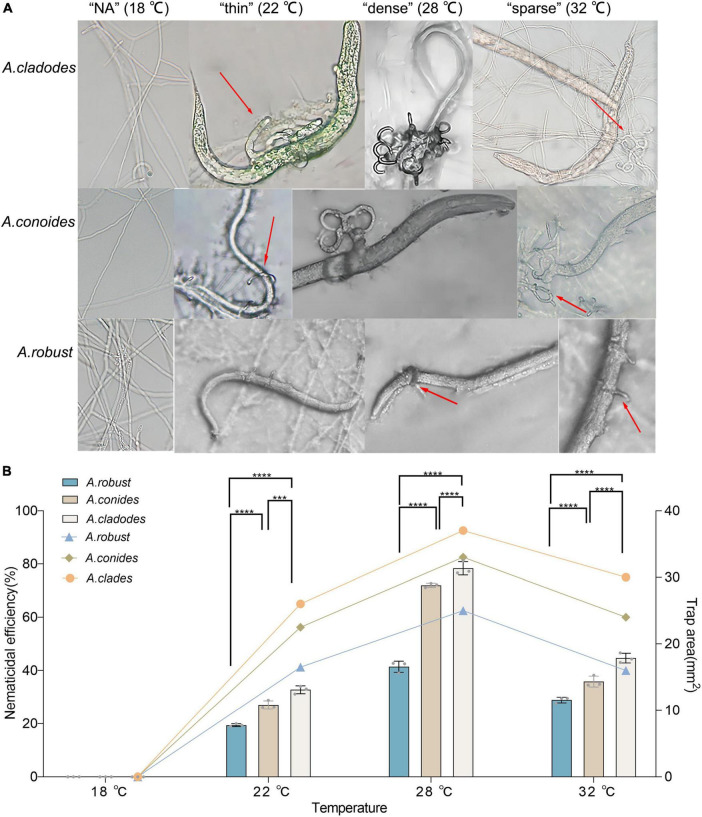
Morphology of the nematicide, nematocidal efficiency, and trap formation area of three nematode-trapping fungi **(A)** Morphology of the nematicide formed by *A. cladodes*, *A. conoides*, and *A. robusta*, corresponding to temperatures **(B)** Nematocidal efficiency and trap area (Histograms indicate nematocidal efficiency and line graphs indicate trap area). Data in figures are the mean SE (*n* = 3), ***P < 0.001 and ****P < 0.0001.

Traps cannot be formed without nematodes. Evaluating whether there are differences in the structure and nematocidal efficiency of traps formed by nematode-trapping fungi under different temperature-stressed environmental conditions induced by the presence of nematodes. The area of the three fungal traps was measured, with temperatures (18, 22, 28, and 32°C) corresponding to the morphology of the different traps, and the nematocidal efficiency was calculated. The mycelium morphology did not change at 18°C due to the inclusion of nematodes, and none of the three fungi could form traps. At 22°C, the mycelium gradually began non-closing three-dimensional structures. However, a few traps were able to form closed rings, which had a “thin” morphology that was very fragile, and adult nematodes easily escaped from the traps. 28°C was considered the most favorable condition for killing nematodes, with trap formation most evident. The formation of “dense” three-dimensional structures means that nematodes are easy to trap and difficult to escape, which is why nematode killing efficiency is relatively high and an important factor that distinguishes them from other conditions. Under unfavorable conditions at 32°C, all three fungi formed “sparse” traps, increasing the difficulty of their capture. The results showed that the area of trap formation, trap morphology, and nematocidal efficiency of different species of nematode-trapping fungi followed the same trend with temperature. The structures of trap formation were NA, thin, dense, and sparse at 18, 22, 28, and 32°C, respectively. *A. robusta*, *A. conoides*, and *A. cladodes* had nematocidal efficiencies of 42.7, 72.18, and 77.2%, respectively, and 25, 33, and 37 mm^2^ areas of trap formation, with *A. cladodes* being comparatively more effective (Figures 2A, B).

### 3.2 Transcriptome analysis

To further investigate the molecular mechanisms of trap formation, we treated *A. cladodes* for 36 h under either nematode-induced or non-induced conditions, based on the temperatures that corresponded to the trap morphology (“NA” −18°C, “thin” −22°C, “dense” −28°C, and “sparse” −32°C). Subsequently, mycelia from 8 *A. cladodes* samples were extracted for transcriptome sequencing, and the sequencing result of each sample was 6.38 Gb. The dataset was deposited in the Sequence Read Archive (SRA; accession: SRP482066; BioProject ID: PRJNA1061069). The error rate was less than 1% with high independence (Supplementary Table 1). To verify the reliability of the transcriptome data, 10 genes were randomly selected for RT-PCR verification. The results showed that the transcriptome data of 10 genes were consistent with the expression trend of RT-PCR data, which indicated that the transcriptome data were reliable (Supplementary Figure 1).

**FIGURE 3:**
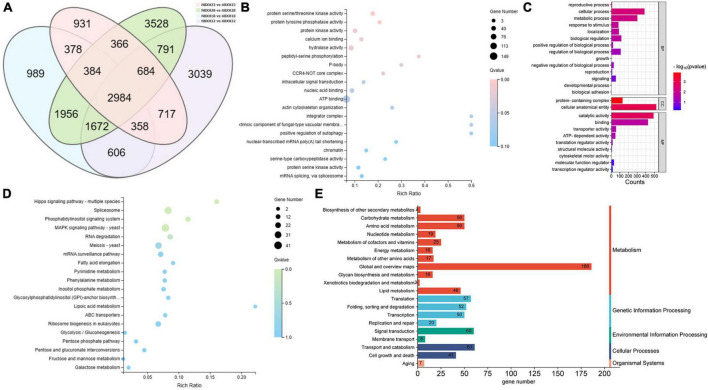
DEGs of temperature stress. **(A)** Venn diagram of DEGs (18, 22, 28, and 32°C) set size represents the number of DEGs. **(B)** GO enrichment analysis **(C)** GO classification **(D)** KEGG enrichment analysis **(E)** KEGG pathway classification of the DEGs (*p* ≤ 0.05).

The intersection of various morphologically differentially expressed genes of the traps was used to screen for 3,528 genes associated with the dense morphology after analyzing the differentially expressed genes in *A. cladodes* infested with PWN under temperature conditions corresponding to the formation of different traps (Figure 3A). Of these, 2,801 genes were upregulated and 727 were downregulated. We performed KEGG and GO enrichment analysis of upregulated and downregulated genes. Nematodes are catabolized and digested during nematode-trapping fungi infesting nematodes and extracting nutrients. Thus, we focused on transport catabolism and hydrolase. Within the category BP, cellular process (GO:0016573), metabolic process (GO:0032259), biological regulation (GO:0000077), response to stimulus (GO:0006979), and within the category MF, catalytic activity (GO:0004674), binding (GO:0005509), and transporter activity (GO:0015078), as well as in CC, genes for cellular anatomical entities (GO:0005634), and protein-containing complexes (GO:0032040) were abundant in induced fungi at 28°C compared to other temperature conditions. ATP binding (GO:0005524), hydrolase activity (GO:0016787), protein serine kinase activity (GO:0106310), and protein kinase activity (GO:0004672) were significantly enriched GO terms according to GO enrichment analysis (Figures 3B, C).

Among the top 20 KEGG pathways, the MAPK signaling pathway (ko04011), pentose phosphate pathway (ko00030), meiosis (ko04113), and ABC transporters (ko02010) were significantly enriched. We focused on the enrichment analysis of the transport and catabolism pathways and screened eight genes (*malz*, *gh18*, *kex2*, *kre6*, *ganab*, *pep4-1*, *ctsd*, and *prb1*) related to it, which were consistent with the trend of the trap structure with temperature. Of these, *malz*, *gh18*, *kre6*, and *ganab* are contained in glycoside hydrolases; *kex2* and *prb1* in serine proteases; and *ctsd*, *pep4-1* in aspartyl proteases. The abundance of trap formation at 28°C was significantly different from the other temperatures (18, 22, and 32°C), as were the genes that were enriched (Supplementary Figure 2).

### 3.3 Key modules associated with the formation of traps

To determine the accuracy of the results, all samples were pooled and filtered for abundance. Low-expression genes were filtered out. Samples were clustered by calculating correlation coefficients for gene expression levels and determining if there were significant outliers. The weight values were calculated using the pickSoftThreshold function in the R package WGCNA to conform the co-expression network to the scale-free network distribution, and the optimal soft threshold β = 8 was determined to generate the hierarchical tree (Supplementary Figure 3).

**FIGURE 4:**
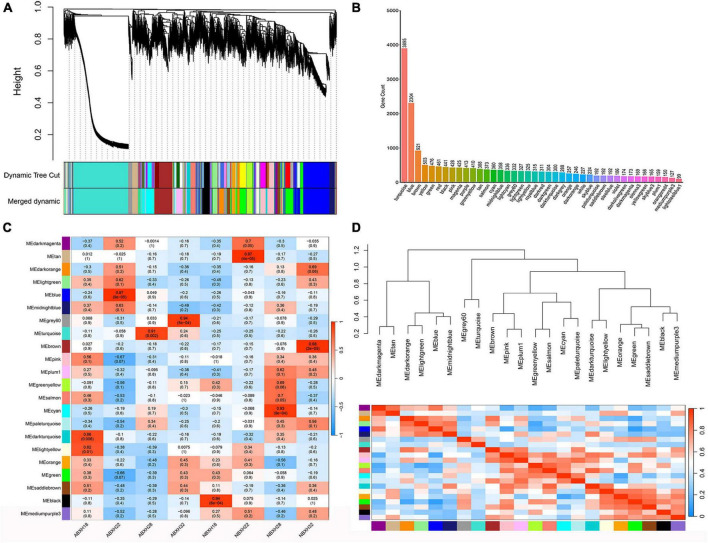
Weighted gene co-expression analysis (WGCNA) showed that modules were highly correlated with traps formation (ABXH28) in *A. cladodes*. **(A)** Clustering dendrograms of genes are constructed based on topological overlap, and different modules are indicated by different colors. **(B)** Distribution of the number of genes in co-expression modules. **(C)** Module-trait associations were estimated using correlations between module eigengenes and different conditions of trap formation. **(D)** Heat map of inter-module correlations.

A clustering tree was constructed based on the correlation of expression among genes, and the resulting clustering tree was cut by using dynamic tree cut and genes with similar expression patterns were merged in the same branch. Then, we merged the modules with similar expression patterns according to the similarity of module feature values to obtain partitioned modules, and different modules were indicated in different colors (Figure 4A). The turquoise module has the highest number of genes, 3895 (Figure 4B and Supplementary Table 3). Gene networks were visualized by drawing heat maps (Supplementary Figure 4).

The correlation between ME and trait was used to identify modules with common expression pattern interactions in the co-expression modules that were linked to specific traits (Figure 4C). The analysis identified two modules (turquoise and gray) associated with traps. Of these two modules, the turquoise module was significantly associated with ABXH28 (correlation value, cor = 0.91, *p*-value = 2 × 10^–3^, Figure 4C), and the gray module was significantly associated with ABXH32 (cor = 0.94, *p*-value = 1 × 10^–4^, Figure 4C). We focus on studies characterized by ABXH28. Relationships between identified modules were investigated by using ME (module signature genes) as a representative profile and quantifying the similarity between modules using ME correlation. The turquoise module had the highest correlation with ABXH28 (Figure 4C), and the dendrograms showed that the turquoise module and gray module were closely related (Figure 4D). This suggests that the morphological similarity of traps is higher under relatively high temperature conditions, as is the similarity of genes in the modules that may function in response to trap formation.

### 3.4 GO and KEGG enrichment analysis of genes in interesting modules

**FIGURE 5:**
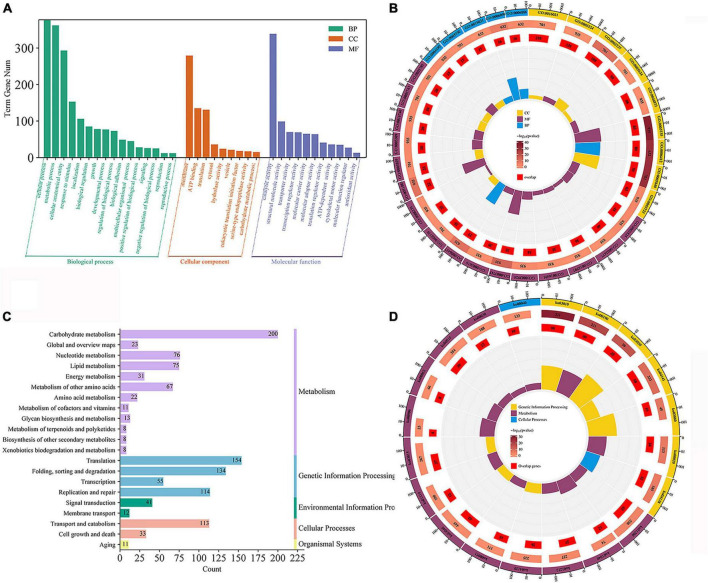
Gene expression patterns of turquoise. **(A)** Go classification; **(B)** GO enrichment circle (*p* ≤ 0.05). **(C)** KEGG pathway classification. **(D)** KEGG enrichment circle (*p* ≤ 0.05).

Gene ontology (GO) enrichment and kyoto encyclopedia of genomes (KEGG) enrichment were performed for genes in the turquoise modules (Figures 5A, B). The genes in this module are mainly enriched in GO: 0016740 (transferase activity), GO: 0016491 (oxidoreductase activity), GO: 0016787 (hydrolase activity), GO: 0005488 (binding), GO: 0005623 (cellular), and GO: 0044464 (cellular fraction). For further functional categorization, the genes in module were mapped to the KEGG database. They were mainly enriched in transport and catabolism (ko04138, ko04145 and ko04136), cell growth and death (ko04113, ko04111), signal transduction (ko04011, ko00620) carbohydrate metabolism (ko00620, ko00910, ko01200) (Figures 5C, D). The above results show that the GO:0016787 (hydrolase activity), translocation and catabolism (ko04138, ko04145, and ko04136) pathways remained significantly enriched compared to genes that were previously upregulated only in ABXH28 (Figures 3B–E).

### 3.5 Interaction network visualization

**FIGURE 6:**
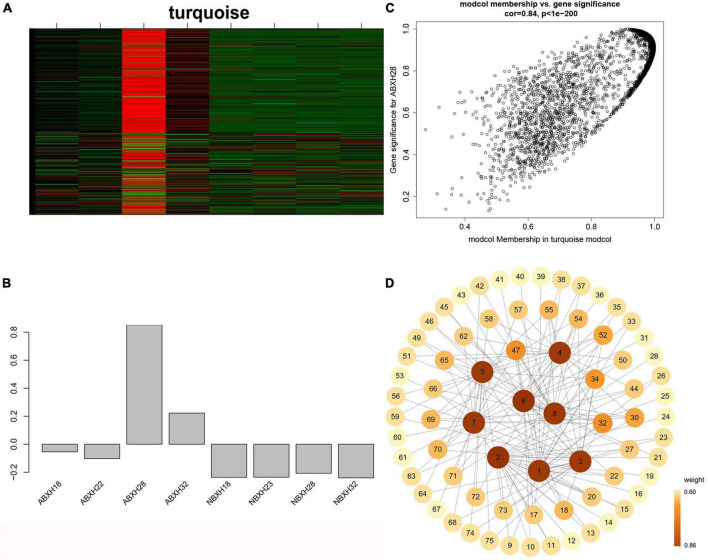
Weighted gene co-expression analysis (WGCNA) reveals gene network modules enriched in *Arthrobotrys clade* ABXH28. **(A)** Heatmap of the turquoise module genes (rows) across the samples (columns) **(B)** Eigengene expression values (*y*-axis) across the samples (*x*-axis) of turquoise module. **(C)** Scatterplot of MM and GS correlations in the turquoise module. **(D)** Gene co-expression network and hub genes in the turquoise module.

We focused on the genetic characteristics of ABXH28, as trapping morphology and nematocidal efficiency were most pronounced at 28°C. The expression of all genes in the turquoise module was compared with the ME expression of the turquoise module in all samples (Figures 6A, B). The results showed that besides the elevated expression in dense traps, the genes in the turquoise module also exhibited significant expression in sparse trap samples (Figures 6A, B). To combine phenotypic characterization information with co-expression networks, gene significance (GS) was defined as the correlation between a gene and a trait to quantify the association between a single gene and the ABXH28 trait. And module membership (MM) was defined as the correlation of the module eigengene and gene expression profiles. GS in the turquoise module exhibited a strong correlation with MM, and genes in the turquoise module (cor = 0.9, *p* = 1 × 1e^–200^) contributed significantly to the biological processes of trap formation compared to other modules (Figure 6C).

**FIGURE 7:**
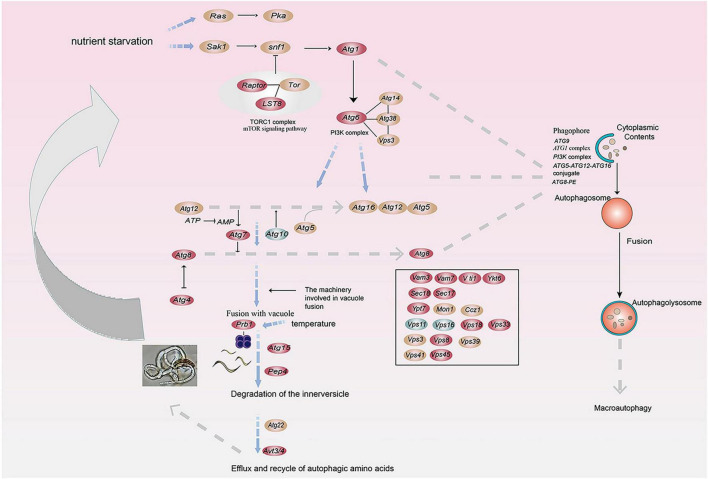
A model for the involvement of the serine protease gene *prb1* in the autophagy pathway in trap formation and nematocidal efficiency by nematode-trapping fungi (red: up-regulated genes; green: down-regulated genes; brown; both up- and down-regulated).

We calculated the weights of each gene in the turquoise module. In this study, eight genes associated with hydrolases and significantly altered with temperature overlapped in two enrichment analyses. Therefore, they are hub genes in modules (Supplementary Table 4). The top 20 genes with the highest weight value of each gene were selected, resulting in 75 genes, which were then numbered (Supplementary Table 4). The network data were exported to Cytoscape (Figure 6D) based on the weight values between two genes (Supplementary Table 5). The entire network contained 160 regulatory relationships for 75 genes (Supplementary Tables 4, 5). The amino acid sequences of 50 of these genes could be compared with highly homologous sequences in the Nr library (Supplementary Table 4). Seven of the eight hub genes (*malz*, *kex2*, *kre6*, *ganab*, *pep4-1*, *ctsd*, and *prb1*) were linked to *pep4-2* in *Rozella allomycis*. six of them (*orc1*, *cdc15-1*, *cdc15-2*, *tor*, *ppp1c*, and *mkk1*) were related to meiosis-related pathways (ko04113, k06683, and ko04111). And *pep4-2* was associated with autophagy-related pathways (ko04138, ko04136), suggesting that these hub genes are closely related to the autophagy and meiosis pathway. According to the KEGG database of autophagy pathways, *prb1* is involved and plays an important role (Figure 7). Thus, we propose a model (Figure 7) that helps to elucidate the molecular mechanisms of genes involved in the autophagy pathway in the formation of nematode-trapping fungal traps and degradation of the nematode body wall.

### 3.6 RT-PCR validation

**FIGURE 8:**
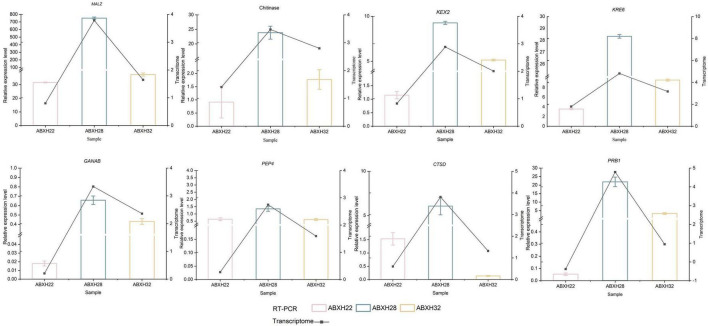
RT-PCR verification of differential genes in ABXH18 vs. ABXH22 (pink), ABXH18 vs. ABXH28 (blue), and ABXH18 vs. ABXH32 (yellow). Histograms indicate RT-PCR (left vertical axis), and line graphs indicate transcriptome (right vertical axis).

The 8 genes mentioned above that are related to hydrolases were selected for further study. Reverse Transcription-Polymerase Chain Reaction (RT-PCR) was used to verify the expression levels of these genes in various trap morphologies (Figure 8). Student’s *t*-test was used to identify significant differences between the normalized expression levels of these genes in ABXH18 and the data. the results showed that the expression levels of the above eight hydrolase-related genes increased and then decreased with increasing temperature. The comparison of gene expression levels between ABXH28 and ABXH22 revealed the biggest difference (*p*-value < 0.0001), with ABXH32 following closely behind. However, there was a significant difference in the expression levels between ABXH22 and ABXH32. Gene expression levels followed the same trend as traps with temperature. At 22°C, gene expression was low, and traps were sparse; at 28°C, gene expression was elevated, and traps were dense; at 32°C, gene expression was reduced, and traps were sparse. Furthermore, the transcriptome results were consistent with the trend of changes in gene expression. The results of transcriptome sequencing were basically consistent, which further proved the reliability of the transcriptome sequencing results.

## 4 Discussion

Nematode-trapping fungi, as a natural enemy of nematodes and a natural nematode antagonist, have received more and more attention and extensive research due to their remarkable environmental friendliness. However, the number of nematodes infested or even killed by nematode-trapping fungi is highly correlated with trap formation, and exceeding the upper and lower temperature limits for fungal growth leads to a decrease in their trapping capacity (Fernández et al., 1999; Sánchez-Martínez et al., 2023). Therefore, it is necessary to investigate the molecular mechanisms underlying the infestation ability of nematode-trapping fungi on nematodes under different temperature conditions. We isolated and characterized *A. cladodes*, *A. robusta*, and *A. conides* from samples in different regions. According to our records, the trend of the area formed by these traps with temperature was relatively consistent. Nematode killing efficiency varies under different temperature conditions, with significant differences in trap morphology. In this study, a morphological comparison of traps at different temperatures was carried out to highlight the differences in trap morphology, and traps with dense structures may be a better choice to differentiate 28°C from other temperatures. Among them, the trap formation of *A. cladodes* was faster and the ability to infest nematodes was more pronounced. We selected *A. cladodes* to further investigate the molecular mechanisms of trap formation.

KEGG and GO enrichment analyses screened for upregulated genes associated with “dense” trap morphology. Since *A. cladodes* degrades the nematode body wall during nematode infestation and draws nutrients for mycelial growth and development, the focus was on transport, metabolic, and hydrolase genes. 8 genes related to hydrolases whose expression varied significantly with temperature were screened.

Then gene characterization of dense traps was carried out using WGCNA to explore the relationship between gene expression profiles and phenotypes to analyze the important relationship between hydrolase-related genes and trap formation. The results indicate that genes highly related to ABHX28 (turquoise module) and ABXH32 (gray module) have similar functions and act in similar pathways. In addition, highly expressed genes in ABHX28 (turquoise module) were also more highly expressed in ABXH32 than in the other stages of *A. cladodes*. However, there were differences in gene expression between ABXH28 and ABXH32, which may be related to a slightly higher temperature induction. Trap morphology and nematocidal efficiency were more similar between ABXH28 and ABXH32 compared to the other stages. These results suggest that these genes may be highly correlated with trap morphology and nematocidal efficiency. Subsequently, we evaluated the genes in the turquoise module, and genes related to hydrolase activity were also significantly enriched, and the eight hydrolase-related genes identified in the up-regulated gene enrichment analyses overlapped with the hydrolase genes enriched in the turquoise module.

Of the 75 genes, 6 are associated with mitosis. In addition, 7 hub genes are linked to *pep4-2*. Moreover, the *pep4-2* gene has been connected to autophagy-related pathways. This suggests that autophagy may play an important role in trap formation and regulate trap formation and nematocidal effects by regulating hydrolase-related genes. Under normal conditions, autophagy occurs at a basal level. However, autophagy is accelerated by various stresses such as nutrient starvation, accumulation of abnormal proteins and organelle damage (Richie et al., 2007; Jiang et al., 2020). Thus far, there is growing evidence that the activation of autophagy appears to be critical for cell differentiation and development (Pollack et al., 2009; Meng et al., 2020). In filamentous fungi, autophagy is involved in mycelial growth, spore germination and pathogenicity of fungi (Liu et al., 2017; Wang et al., 2019; Jacomin et al., 2020). In nematode-trapping fungi, autophagy is involved in cell growth and trap formation (Chen et al., 2013). In addition, hydrolytic enzymes are required in the autophagy pathway to involve in and break down the products of the fusion of autophagosomes with vesicles, yielding metabolites that are reused (Veenhuis et al., 1989), suggesting that autophagy and hydrolytic enzymes are tightly correlated.

The combination of transcriptomic and qPCR differential genes in the autophagy pathway suggests a model for predator formation in *A. cladodes*, i.e., that *A. cladodes* initiates a complex series of cellular responses upon nutrient starvation. Among them, the serine protease gene prb1 is directly involved in the autophagy pathway and is converted to protease A, which has the capability to hydrolyze proteins (Moehle et al., 1988). In addition, the nematode body wall is composed of proteins (Cohen and Sundaram, 2013), and serine proteases digest and break down nematodes to provide nutrients for cell growth (Li et al., 2010). The differences in temperature lead to differences in the rate at which hydrolytic enzymes such as serine proteases break down and digest the nematodes, which affects the uptake of nutrients by the mycelium, and finally the growth of the cells and the degree of sparseness of trap formation. In the present study, the expression of hydrolases was significantly increased during the nematode-trapping fungal trap formation and nematocidal stages, which further indicates the important role of hydrolases in this stage. There is growing evidence that hydrolytic enzymes are involved in fungal growth and development and pathogenicity, such as *prb1* (vesicular protease B) as well as glycoside hydrolases. prb1 is a serine protease found to have significant hydrolase activity in yeast (Moehle et al., 1988). In rice, it regulates growth and development by degrading the transcriptional co-repressor *OsTPR2* and affecting the level of histone acetylation in the phytohormone signaling pathway (Huang et al., 2004; Li et al., 2023). Glycoside hydrolases are involved in the regulation of the growth and pathogenicity of the fungus *Verticillium dahliae*. It shows sensitivity in response to abiotic stresses and influences the pathogenicity of *V. dahliae* on cotton (Wen et al., 2023). In general, autophagy plays a very important role in mycelial growth, spore germination, and the pathogenicity of filamentous fungi (Liu et al., 2017; Wang et al., 2019; Jacomin et al., 2020).

In previous transcriptomic studies of nematode trapping fungi, the molecular mechanisms involved in the complex interactions of the nematode infestation stage of nematode trapping fungi originating from soil were mainly addressed (Li G. et al., 2007; Li L. et al., 2007; Zhu et al., 2022). Such as transcriptome analysis of *Arthrobotrys oligospora*, *Monacrosporium cionopagum*, and *Arthrobotrys dactyloides* infested with two plant-parasitic nematodes and showed that the differences in gene expression between the fungi were significantly greater than the differences between the fungi infested nematodes (Andersson et al., 2014). Transcriptome analysis of the molecular mechanisms of Arthrobotrys conoides and *Duddingtonia flagrans* infestation of the root-knot nematode stage and *Monacrosporium haptotylum* infestation of the *Caenorhabditis elegans* stage, among others (Fekete et al., 2008; Pandit et al., 2017). In this study, we isolated nematode-trapping fungi from diseased trees and had targeted infestation of *B. xylophilus*. Based on the observation that temperature significantly affects its nematocidal efficiency and traps formation, we performed transcriptomic analyses to investigate the important role of temperature in the mechanism of its infestation of pine wood nematodes and to analyze the genes related to the infestation stage in which nematode-trapping fungi have a greater nematocidal efficiency and traps formation capacity. Associating nematode-trapping fungi with temperatures that cannot be artificially regulated in complex and variable natural environments lays the foundation for the application of nematode-trapping fungi to future forest control.

Its nematocidal activity may be influenced by two factors. Morphologically, it’s evident that the nematocidal efficiency is positively correlated with the formation of traps, meaning that the more traps are formed, the stronger the insecticidal activity becomes. Molecular mechanisms show that the expression levels of hydrolase-related genes correspond with the trends in trap formation as temperatures vary. At 28°C, there is a significant expression of enzyme-producing genes in the “dense” traps, whereas in the “thin” and “sparse” traps, the expression levels of these enzyme-producing genes are comparatively lower. Therefore, we believe that hydrolases play a crucial role in the formation of traps and in the process of killing nematodes. In addition, the chemical composition of the host body wall barrier reflects the types of extracellular hydrolases used by plant and animal parasitic fungi in penetrating and infesting the host, which may help to elucidate the fungus-host interaction (Huang et al., 2004). Based on transcriptomic data, we found that *prb1* is involved in the autophagy pathway of *A. cladodes*. Therefore, we propose a model in which temperature affects trap formation and nematicidal efficiency by influencing prb1, which is involved in the autophagy process.

In the natural environment, *B. xylophilus* has a relatively strong ability to reproduce at 28°C. Meanwhile, we found that the nematocidal efficiency of nematode-trapping fungi was relatively significant at 28°C. The application of nematode-trapping fungi can be considered by choosing the temperature under the relatively strong reproductive ability of *B. xylophilus* to achieve the maximum effect of control. Temperature affects the ability of hydrolase genes to produce enzymes, the structure of traps, and the ability of nematocidal activity. Therefore, selecting the appropriate temperature range is critical when evaluating the long-term control effects of nematode-trapping fungi on *B. xylophilus*. In practical applications, it is essential to thoughtfully consider and select the appropriate temperature conditions to ensure that nematode-trapping fungi achieve optimal efficacy in controlling *B. xylophilus*. In addition, we should actively investigate gene editing to regulate the expression of hydrolase genes in future experiments to improve the pathogenicity and virulence of nematode-trapping fungi, aiming to maximize the potential of *A. cladodes*.

## Data availability statement

The datasets presented in this study can be found in online repositories. The names of the repository/repositories and accession number(s) can be found in the article/Supplementary material.

## Author contributions

HJ: Conceptualization, Data curation, Formal analysis, Investigation, Methodology, Resources, Software, Validation, Visualization, Writing – original draft. RX: Writing – review & editing. RZ: Writing – review & editing. GL: Methodology, Validation, Writing – review & editing. YuZ: Investigation, Writing – review & editing. YaZ: Project administration, Supervision, Writing – review & editing. DL: Conceptualization, Project administration, Supervision, Writing – review & editing. FW: Funding acquisition, Project administration, Supervision, Writing – review & editing.

## Footnotes

1
https://github.com/bgi-flexlab/soapnuke

2
http://bowtie-bio.sourceforge.net/bowtie/index.shtml

3
https://github.com/deweylab/RSEM
