# Supplementary material for: Transcriptome analysis highlights the influence of temperature on hydrolase and traps in nematode-trapping fungi
Source: Front Microbiol. 2024 May 7;15:1384459. doi: 10.3389/fmicb.2024.1384459 (PMC11106486; doi:10.3389/fmicb.2024.1384459)
Supplement: Supplementary file 1 [file Data_Sheet_1.PDF]

Supplementary Material

1 Supplementary Figures and Tables

1.1 Figures

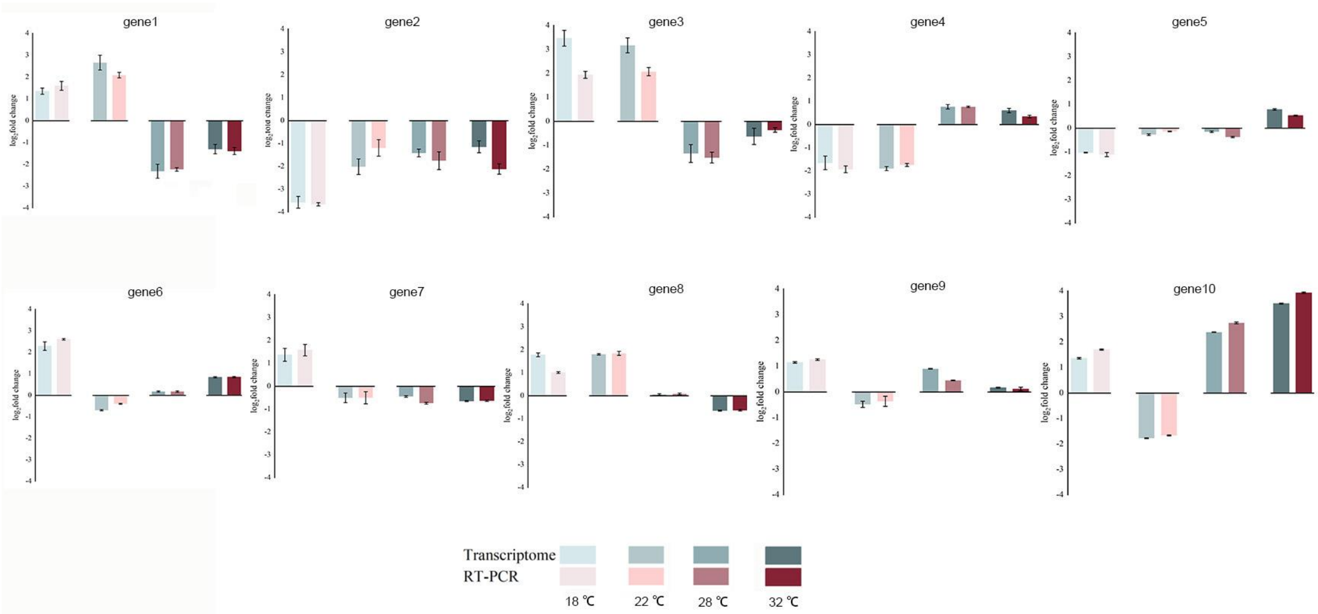

Figure S1. RT-PCR verification of DEGs in the transcriptome

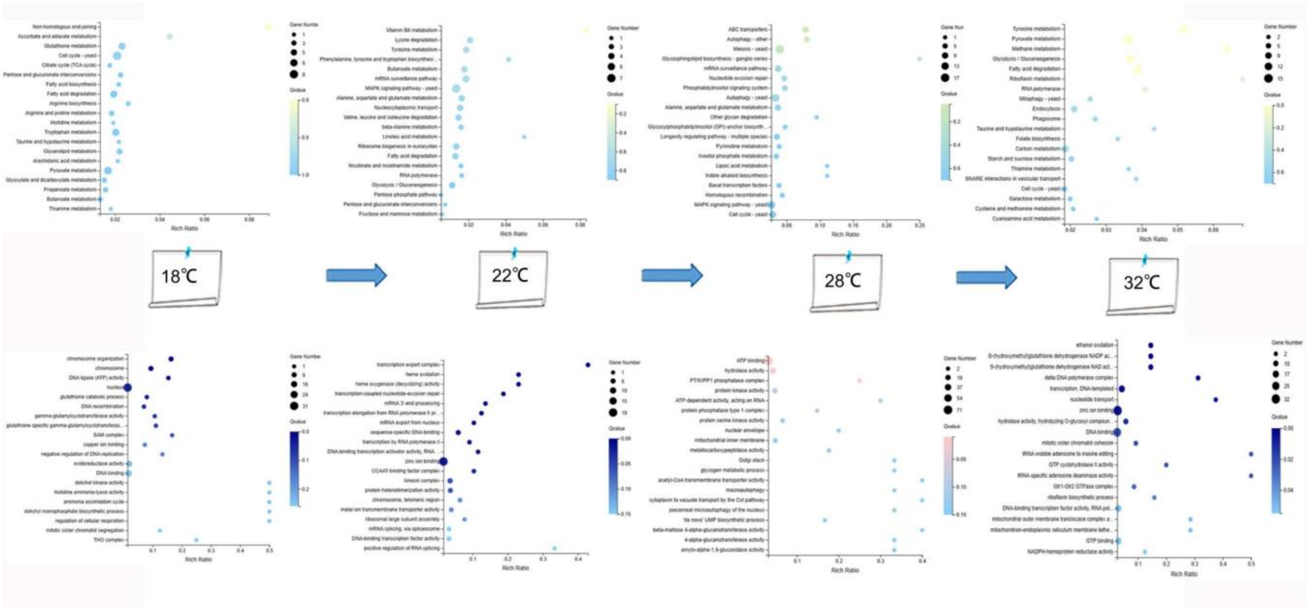

Figure S2. DEGs of *A. cladodes* induced by *B. xylophilus* under different temperature conditions were analyzed by KEGG and GO. KEGG enrichment was analyzed at 18°C, 22°C, 28°C, and 32°C (A, B, C, D), and GO was analyzed at 18°C, 22°C, 28°C, and 32°C (E, F, G H).

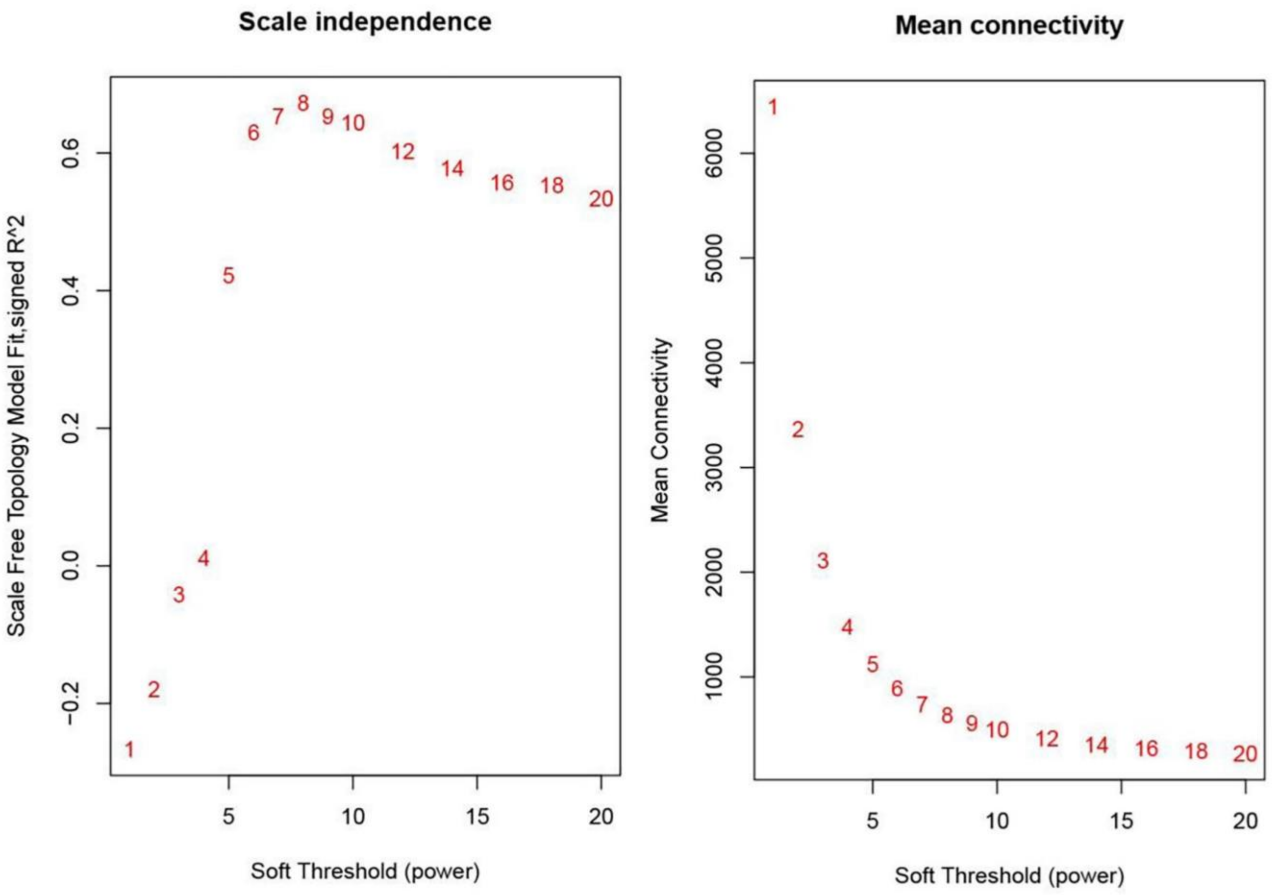

Figure S3. Co-expression network analysis. The left figure shows the scale-free fit index (y-axis) as a function of the soft threshold power (x-axis). The right figure shows the variation of average gene connectivity under different power.

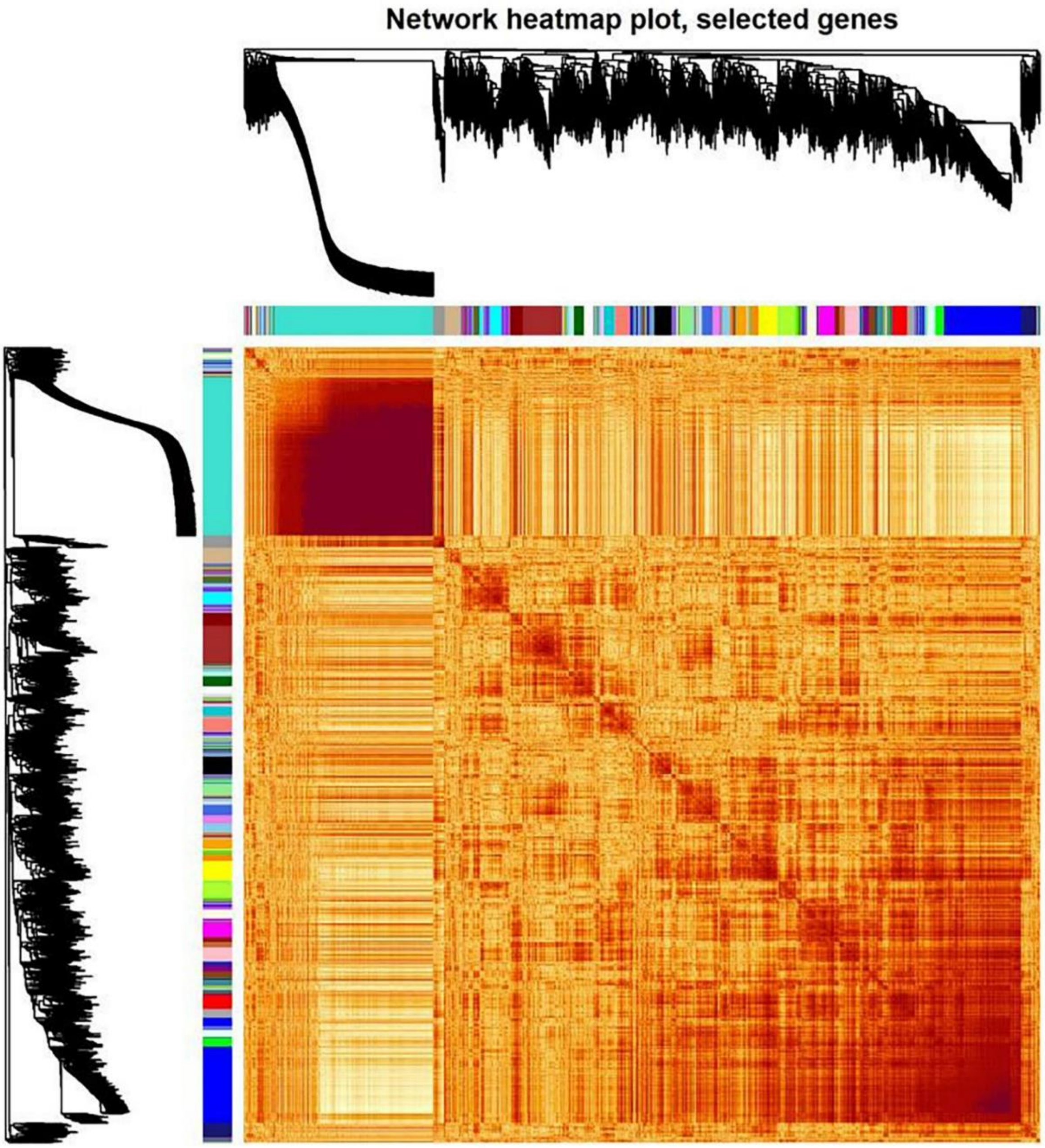

Figure S4. Heat map of module gene clustering

1.2 Tables.

Table S1. Statistics analysis of RNA sequencing data

Sample name	Total raw reads (Mb)	Total clean reads (Mb)	Total clean bases (Gb)	Clean reads Q20(%)	Clean reads Q30(%)	Clean reads ratio (%)
ABXH18	43.69	42.51	6.38	98.69	94.77	97.3
ABXH22	43.69	42.5	6.38	98.61	94.39	97.28
ABXH28	43.69	42.54	6.38	98.67	94.64	97.36
ABXH32	43.69	42.55	6.38	98.51	94	97.4
NBXH18	43.69	42.42	6.38	98.68	94.67	97.1
NBXH22	43.69	42.21	6.38	98.66	94.64	96.6
NBXH28	43.69	42.54	6.38	98.58	94.27	97.37
NBXH32	43.69	42.75	6.38	98.61	94.44	97.85

Table S2. RT–PCR Primers used in this experiment.

Gene	Forward primer	Reverse primer
malz	GTGCTCCACATGGCATTCA	TTATTTGGCGTCGAGCACT
gh18	TTATTTGGCGTCGAGCACT	TTAGGCCAACGCCTTCTTGA
kex2	GTCCACAAAATGCCAACCA	CTACTTCCACAGGTCCAGCA
kre6	ATGGAGACATACGATTCGA	CTATTGCTCCAAGTAGGGCT
ganab	ATGTTTATCCCATCTCACGA	TTAGGAGTCGTCCTCCGGCA
pep4	CTCCGACAACTTTTGTTGGGA	GGTGTAATCGTAGATCTGGCA
ctsd	AGAAAATTCTGGGTGCAGA	TTACAACTTGCCCATCAAGAT
prb1	ATGAAGTCCTTCACTTTTA	TTACAACTTTTGAAGCTGTT
18S	GGAACACCTTTACAGCACAAAT	TGCCAAACGTAGCAATCCAT
β-actin	TCGATGGCTCCGGTGTTTAA	TTGTAATGACCCTTCGCCCA
gene 1	ATGGATCCGCCACAAAATCCCG	TTAAAGGCCTAGTGATTGCAA
gene 2	ATGAGATCCAGGTCCTTCCT	ATTCCATAGAATACCCAGT
gene 3	GTCAAATCTCTCATCGCCG	CAGTAATCCGGAAGCAACGA
gene 4	GATTCTGATACCGATATCGTT	CCCGACGATGGATCCTAAGAG
gene 5	GACCGAGCAAGAAGGGGGT	TCAGGTCATCAGCACCTGCA
gene 6	TCGTCTGTCGACTCCGTCCCT	TCAGGTCATCAGCACCTGCAA
gene 7	ATGATGGATCCATATTCCTTT	TTAACCATTTATGGAGCCA
gene 8	TCTATACCAGACTCAACAT	AGATTTTTGTAAGTTTGTGAA
gene 9	TCTATACCAGACTCAACAT	AGATTTTTGTAAGTTTGTGAA
gene 10	ATAACAATCGATCTTCAATTTG	ATGTAACAATATTTGCCGGA

Table S3. Number of genes in each module

Module colors	Gene number
turquoise	3895
blue	2304
brown	921
yellow	503
green	476
red	461
black	441
pink	428
magenta	425
purple	413
greenyellow	410
san	388
salmon	373
cyan	360
midnightblue	358
lightcyan	336
lightgreen	327
grey60	332
lightyellow	325
royalblue	315

drakturquoise	300
darkred	311
darkgreen	304
drakgrey	288
drakorange	246
orange	257
white	227
skyblue	224
paleturquoise	192
saddlebrown	192
steelblue	192
violet	186
darkolivegreen	174
darkmagenta	173
sienna3	169
yellowgreen	166
skyblue3	165
plum1	159
orangered4	150
mediumpurple3	127
lightsteelblue1	99
